# Quantitative Speech Assessment in Ataxia—Consensus Recommendations by the Ataxia Global Initiative Working Group on Digital-Motor Markers

**DOI:** 10.1007/s12311-023-01623-4

**Published:** 2023-10-28

**Authors:** Adam P. Vogel, Anna Sobanska, Anoopum Gupta, Gessica Vasco, Marcus Grobe-Einsler, Susanna Summa, Stephanie Borel

**Affiliations:** 1 Centre for Neuroscience of Speech, The University of Melbourne, Melbourne, Australia; 2 Division of Translational Genomics of Neurodegenerative Diseases, Hertie Institute for Clinical Brain Research, University of Tübingen, Germany & Center for Neurology, University Hospital Tübingen, Tübingen, Germany; 3 Redenlab Inc., Melbourne, Australia; 4 Department of Clinical Neurophysiology, Institute of Psychiatry and Neurology, Warsaw, Poland; 5 Department of Neurology, Massachusetts General Hospital and Harvard Medical School, Boston, MA USA; 6 Bambino Gesù Children’s Hospital, IRCCS, 00050 Rome, Italy; 7 German Center for Neurodegenerative Diseases, Bonn, Germany; 8 Department of Neurology, University Hospital Bonn, Bonn, Germany; 9 Sorbonne Université, Paris Brain Institute (ICM Institut du Cerveau), AP-HP, INSERM, CNRS, University Hospital Pitié-Salpêtrière, F-75013 Paris, France

**Keywords:** Speech, Dysarthria, Patient-reported outcome, Clinical trials, Acoustics

## Abstract

Dysarthria is a common and debilitating symptom of many neurodegenerative diseases, including those resulting in ataxia. Changes to speech lead to significant reductions in quality of life, impacting the speaker in most daily activities. Recognition of its importance as an objective outcome measure in clinical trials for ataxia is growing. Its viability as an endpoint across the disease spectrum (i.e. pre-symptomatic onwards) means that trials can recruit ambulant individuals and later-stage individuals who are often excluded because of difficulty completing lower limb tasks. Here we discuss the key considerations for speech testing in clinical trials including hardware selection, suitability of tasks and their role in protocols for trials and propose a core set of tasks for speech testing in clinical trials. Test batteries could include forms suitable for remote short, sensitive and easy to use, with norms available in several languages. The use of artificial intelligence also could improve accuracy and automaticity of analytical pipelines in clinic and trials.

**Supplementary Information:**

The online version contains supplementary material available at 10.1007/s12311-023-01623-4.

## Introduction

Ataxia leads to changes in speech [1–6]. These changes worsen as the disease progresses [7] and can improve with effective treatment [8–10]. Subtle changes can even occur prior to disease onset [3]. Broadly, the ataxia speech phenotype is characterised by a reduced rate of speech, imprecise production of consonants, distorted vowels [1, 11], dysphonia [12] and hypernasality [13]. The dysarthric profile also includes poor vocal control (incoordination of pitch and loudness) and diminished breath support [14]. These deficits, in part, are the result of mis-timed and inaccurately targeted articulatory movements resulting in slower and slurred speech [15, 16]. Combined, dysarthria resulting from ataxia impacts naturalness and intelligibility. Dysarthria can lead to daily disadvantage and prevent simple communication exchanges from occurring (e.g. signalling food preferences, need for toileting). It can also trigger altered self-identity [17] and impede or prevent both social and professional interactions [18], leading to daily disadvantage, producing social marginalisation [19] and underemployment [18]. Seventy percent of people with a communication disorder are unemployed or in the lowest income brackets [20].

Despite the debilitating daily impact of dysarthria, objective measurement of speech is rarely addressed in ataxia clinical practice, clinical trials and research. This may be due to the relatively limited influence of speech on overall scores in commonly used disease severity scales (i.e. SARA, ICARS, mFARS) [21]. On the other hand, speech is considered key feature for measurement in patients (https://www.ataxia.org/ataxiapfdd/), becoming the most important quality of life when individuals become non-ambulant [22, 23]. When it is examined, published cohort studies are often small and restricted to specialised centres which are informative, but their generalizability is limited. There also is limited published longitudinal and natural history data and inadequate evidence-based interventions for speech [24, 25]. In clinical disciplines involved in managing ataxia, decisions about disease-related dysarthria are mainly based on subjective assessment of speech symptoms. Yet a strong body of evidence has consistently shown that more precise speech measurement can increase sensitivity of clinical decisions and provide greater information on the nature of neurological change as well as determining the potential benefits of pharmaceutical and behavioural therapies aimed at forestalling symptomatic progression [9, 10].

**Fig. 1:**
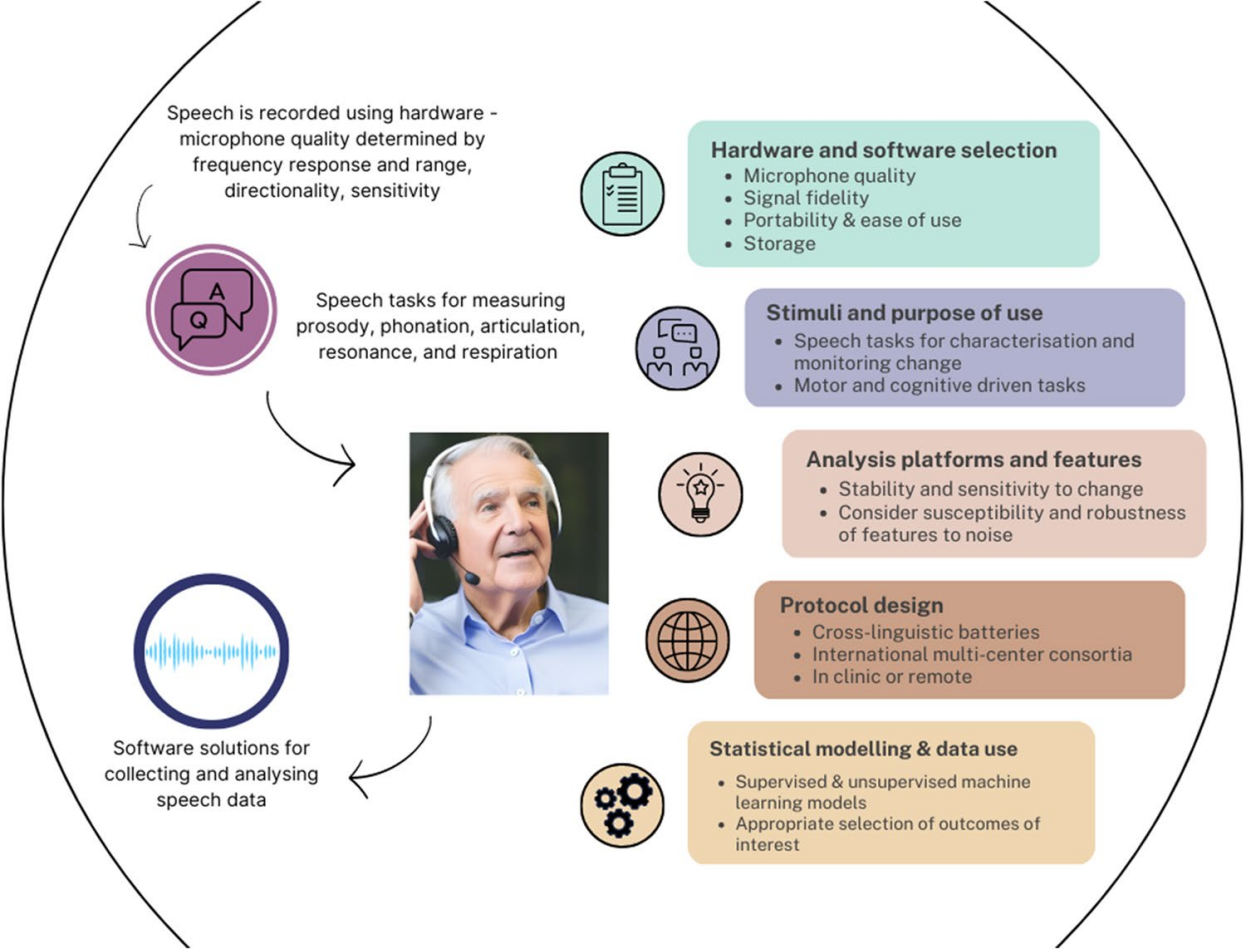
Considerations for using speech as a clinical outcome measure

For over a century, speech disorders have been described by what the listener can subjectively hear, despite early attempts at quantification [26]. Advances in signal processing, cloud computing and hardware and remote data capture provide an opportunity to exploit the intrinsic utility of speech as a marker of disease progression and treatment response (see Fig. 1). Digital technologies have the potential to surpass clinical judgement for accuracy and accessibility as they can yield objective outcomes and can be administered in the clinic or home. Here we outline considerations for use of speech as a marker of performance and quality of life in clinical trials. We also provide recommendations for protocol design, hardware and software selection, features of importance for describing change and disease state, links to patient reported outcomes, existing datasets and ongoing natural history studies.

### Hardware and Software Selection

Audio files are typically recorded and stored for post testing analysis^1^. A microphone is used to capture speech and it is an important determinant of signal quality. Microphone quality and suitability for recording speech is determined by its frequency response and range, directionality, polar pattern and power supply (see [27, 28] for details). Signal fidelity is also influenced by file format, sampling rate, physical elements of the device and noise. Captured audio can be stored in a lossless format (e.g. .wav), preserving all aspects of the signal within the predetermined sampling rate. Elements of speech important for communication fall within the first 5KHz, with a minimum suggested sampling rate twice the maximum frequency of interest [29]. Thus, to ensure adequate fidelity, it is recommended that files be sampled at a minimum of 16KHz, 8-bit quantization with post recording down-sampling completed if necessary. Noise can enter the signal through several sources including the environment (e.g. other speakers, air conditioning), low-quality or poorly insulated wiring, or inappropriate positioning of the microphone (e.g. too close/far, or variable distance to source). Portability, ease of use and budget also guide decisions about utility of recording set ups (see [30–32] for example comparisons between devices).

Specific hardware recommendations are not included in this review as technology is constantly evolving; each study or testing environment will likely benefit from a configuration tailored to the specific use case. For example, for large community samples, a bring-your-own device may be selected by investigators, or similarly, provisioned portable devices may be selected for use in a clinical trial that only interact with specific microphone solutions. Readers are encouraged to read published comparative studies [30–32] or tutorials [27, 28] for more information to assist with hardware selection.

Software, apps and digital interfaces used to capture speech should allow for modification or set minimum standards when collecting audio. A significant proportion of speech capture software now provides cloud storage of audio rather than on-device storage. Remote storage can assist in data dissemination, access to analytic platforms and secure storage. It is recommended that systems used to capture and store audio encrypt data at rest and in transit are not directly identifiable beyond the audio file itself and meet multi-region privacy regulations outlined in the Health Insurance Portability and Accountability Act (HIPAA) and Europe’s General Data Protection Regulations (GDPR).

### Stimuli and Use

Speech protocols should be theory driven and influenced by strong empirical evidence supporting their use. The motivation for testing also shapes protocol design. For example, assessment for characterisation is not necessarily well suited to detecting change from treatment [33, 34]. Characterisation of speech deficits requires an in-depth investigation describing specific impairments (e.g. voice quality), their impact on function (e.g. intelligibility) and their influence on participation (e.g. quality of life). Characterisation protocols may include speaking in a variety of contexts, across multiple tasks, and include listener ratings alongside patient reported outcomes. Batteries that support investigation of key speech domains of prosody, voice, articulation, resonance and respiration are appropriate for phenotyping studies. Tasks could focus on connected speech (e.g. conversation) to assess articulation, prosody and resonance, and challenge activities such as diadochokinesis (DDK) (i.e. PATA) for timing, coordination and articulation, and maximum phonation time (MPT) (for breath support and voice quality). Maximal challenge tasks such as MPT or DDK and oral motor mobility tasks such as those in cranial nerve exams may be appropriate to measure performance across severity levels. They can test a speaker’s maximal abilities, provide data on severity and are independent of language. Beyond singular measures of severity, global features like intelligibility and naturalness of spontaneous speech bring together information on all speech subsystems and are a strong reflection of daily life difficulties. Measures of intelligibility can be derived via standardised clinician perceptual scores or via composite measures of multiple acoustic features. Speech to text tools can also provide an estimate of intelligibility; however, these estimates are dynamic as they are built on models that are constantly evolving. It is possible to rely on these tools in circumstances where measures are based on a specific version of the model; that model can be “frozen” for persistent use [35]. Accuracy of speech to text models also vary based on sex, accent, age and language of the speaker [36].

Testing for the purpose of detecting change in performance can be achieved through a brief, easy-to-administer and complete battery that is motivating and provides capacity for comparison over multiple time points (see [34] for discussion on tracking change in speech studies). Performance should remain stable in the absence of true change, and change when central nervous system function is compromised, through disease or physiology (e.g. fatigue) [37].

We know that speech is sensitive to disease in ataxia (see Table S1 for exemplar studies); however, it is rare for other influencing factors to be considered in study design. Speech changes with fatigue [37], repeated application [33], depression [38], altered feedback [34], the role of the assessor [39], the duration of the sample [40], phonetic context [41] and emotional states like boredom [42]. The influence these factors exert on speech production highlights the need for informed protocol design when the aim is monitoring change. Further, recognition that cerebellar disorders can lead to concomitant cognitive deficits [43] alongside motor dysfunction dictates the need for speech protocols to include simple, brief tasks that fit along a continuum of motor/cognitive complexity [44]. A similar model of assessment has been applied to other neurodegenerative diseases with motor and cognitive decline (e.g. Huntington’s disease [45] and Fronto-temporal dementia [46]). Protocol establishment should be developed alongside intrinsic properties of methods for analysing data and the features they yield. These include listener-based judgement, standardised assessments for measuring aspects of speech (e.g. [47]), instrumental assessments (e.g. electromagnetic articulography) or acoustic analysis.

### Analysis Platforms and Features

To establish the suitability of tasks (and analysis algorithms) for tracking change, they should be subjected to both stability and sensitivity challenges [33, 41]. Stability can be evaluated by eliciting speech repeatedly over brief and extended inter-recording intervals. This is designed to examine susceptibility and robustness of tasks and features to change. It is important to interrogate error or noise arising from technological issues relating to equipment or biological change like diurnal variability, altered motivation or fatigue. Following establishment of task and feature stability (the absence of change), sensitivity needs to be considered. Tasks and features may be stable because they are truly robust to noise, or they may simply be insensitive to change and therefore unsuitable for tracking change. Sensitivity can be estimated through challenges like sustained wakefulness [37], noise [44] or disease itself compared to a norm [5, 48].

Unlike a decade ago, there are now a plethora of software solutions for collecting and analysing speech data. When selecting appropriate digital resources for speech, there are data security, quality and usability features to consider. Ensure data are secure, encrypted at rest and in transit, are not stored alongside any personally identifiable information and are not altered (e.g. compressed) before storage. If using normative data provided by a software provider, check its veracity and suitability for comparison with your own dataset. There are reputable software options available from academic and commercial entities as well as normative datasets.

### Protocol Design

Batteries for the assessment of dysarthric speech have been developed in some languages, such as the MonPaGe battery (French) [48] and the Bogenhausen Dysarthria Scales (BoDyS) (German) [49]. But the challenge for improving research in ataxia is now to develop trans-linguistic batteries that can be used as biomarkers in international multicentric studies. Such protocols include language-independent tasks like prolonged vowel production and syllable repetition. Although there is considerable overlap between sites, investigators and batteries, the ad hoc approach to study design for each study or each language do not allow for multi-centre or inter-pathology comparison. The scientific and clinical community need to develop all together a core of the protocol that would be short, sensitive and easy to use, with norms available in several languages. There are some exemplar initiatives bringing protocols together including the SpeechATAXIA project established within the Ataxia Global Initiative (https://ataxia-global-initiative.net/projects/speech-ataxia-a-multinational-multilanguage-consortia-for-speech-in-hereditary-ataxias/), the Friedreich ataxia Clinical Outcomes Study (FA-COMS) run by the Friedreich's Ataxia Research Alliance (https://www.curefa.org/clinical-trials-active-enrolling/clinical-outcome-measures-in-friedreich-s-ataxia-a-natural-history-study) and the new FA Global Clinical Consortium (FA-GCC) which combines FA-COMS and EFACTS (the European Friedreich’s Ataxia Consortium for Translational Studies).

Speech studies can be run face to face in the clinic or remotely at home. Data can be collected on specialised audio equipment or consumer grade devices. Users can bring their own device (BYOD) to studies or use provisioned setups where hardware are provided by investigators. BYOD and remote testing can be advantageous in some settings and may provide freedom of users to complete tests when and where they choose. There is also an ability for investigators to collect data in what is perceived to be more ecologically valid testing conditions, such as in the home during daily activities. The latter leads to legitimate concerns around privacy and data use. Out-of-clinic recordings can also be hindered by reduced sound quality through non-provisioned devices or background noise for example.

### Potential Application of Machine Learning and Data-Driven Statistical Models

Artificial intelligence (AI) and big data analysis are methods that may enhance our ability to identify symptom onset or monitor disease progression in ataxia. Attempts to expand its use in diagnosis are underway [50]. The purpose is not to consider each single digital parameter as a biomarker but to define all the relevant information contained in the speech signal and use them as a parsimonious subset as determined by machine learning (ML) and deep learning (DL) algorithms. Learning feature representations is a central tenet of deep learning—the model can learn patterns directly from the audio time series that are informative for downstream tasks such as disease classification or severity estimation. Machine learning models can be trained in a supervised or unsupervised manner. In supervised learning, the sample data is already labelled, and it is used to train a classification or regression model. Then unlabelled data is given to this trained system for labelling (e.g. classify) based on the features. In unsupervised learning, the training set is not labelled. The system itself learns the structure of the data, for example to identify clusters or latent factors. In both the methods, selection of features plays an important role, as does the sample data dimension used to train the model. There are some ML studies seeking to separate ataxic speakers from healthy speakers [51–54]; however, the value of this exercise is diminished by the knowledge that ataxia is a multi-faceted disease group requiring multi-modal assessments for diagnosis. An alternative or additional and potentially more valuable use of ML for speech draws on communication outcomes that are meaningful for patients and clinicians, such as intelligibility and naturalness [55]. This approach treats speech as an outcome in its own right, in addition to its role as a subcomponent of a diagnostic workup. In addition to those papers cited, we can gain insight into AI utility from other neurological disorders with similar symptoms [36, 54, 56]. As mentioned, binary or ternary classifiers are commonly used to distinguish between the healthy and pathological conditions [56, 57]. Often these discriminative models apply very simple feed-forward artificial neural networks (ANN) and super vector machines (SVM) [56, 57]. There are also studies that use binary or ternary classifiers to discriminate different levels of dysarthria severity using the Mahalanobis distance and reaching 95% or higher accuracy in splitting groups [51]. Other binary algorithms examples are linear discriminant analysis (LDA) and k-nearest neighbour but with lower accuracy [54]. As is the case with other behavioural markers, adding sensitivity beyond binary outcomes (e.g. adding levels of intelligibility) can lead to decreases in accuracy [58]. Some recent examples of hierarchical machine-learning model (combination of machine and deep learning algorithms) revealed promising results in ataxic groups [55, 59–61]. It is reasonable to assume AI will have role in future clinical practice, but it is important to understand its current limitations; for example, AI requires suitable and sufficient data [57].

## Conclusion

Speech disorder caused by hereditary ataxia triggers altered self-identity, and impedes social and professional interactions, leading to daily disadvantage, producing social marginalisation and underemployment. These changes typically worsen as the disease progresses but may improve with treatment. Subtle changes can even occur prior to diagnosis. The centrality of speech in daily life highlights its importance in clinical care and as a marker of brain health. We have provided clear information on the practical and theoretical factors driving protocol design, data collection, features of interest and links to meaningfulness for stakeholders.

### Supplementary Information

ESM 1: Table S1. Exemplar studies describing associations between speech and clinical measures of disease. (DOCX 39 kb)

## Data Availability

Not applicable.

## Footnotes

1 There are circumstances where a microphone is not used (e.g. electroglottography) and where data are not stored on the device (e.g. contemporaneous analysis of filtered components of the waveform or spectrogram, on device).
