# Supplementary material for: Quantitative Speech Assessment in Ataxia—Consensus Recommendations by the Ataxia Global Initiative Working Group on Digital-Motor Markers
Source: Cerebellum. 2023 Oct 28;23(3):1128–34. doi: 10.1007/s12311-023-01623-4 (PMC11102369; doi:10.1007/s12311-023-01623-4)
Supplement: Supplementary file 1 — Table S1. Exemplar studies describing associations between speech and clinical measures of disease. (DOCX 39 kb) [file 12311_2023_1623_MOESM1_ESM.docx]

Supplementary Materials

Table 1: Exemplar studies describing associations between speech and clinical measures of disease.

| **Authors** | **Ataxia** | **N** | **Speech domain or task** | **Clinical/genetic evaluation** | **Correlation (r / rho) or Mean Adjusted Error (MAE)** |
| --- | --- | --- | --- | --- | --- |
| Eigentler et al., 2012 (1) | FA | 15 | Frenchay Dysarthria Assessment | GAA1 | R = -0.768 |
| Eigentler et al., 2012 (1) | SCA2, 6, 28 | 7 | Frenchay Dysarthria Assessment | SARA | R = 0.932 |
| Vogel et al., 2018 (2) | ARSACS | 11 | Frenchay Dysarthria Assessment (intelligibility) | Disease duration | R = 0.66 |
| Singh et al., 2010 (3) | FA | 22 | Oral motor examination (motor and word repetition) | FARS | R = -0.73 |
| Borel et al., 2019 (4)^b^ | FA | 40 | Oro-facial mobility | SARA | R = -0.63 |
| Borel et al., 2019 (4)^b^ | FA | 40 | Oro-facial mobility | CCFS | R = 0.67 |
| Borel et al., 2019 (4)^b^ | FA | 40 | Oro-facial mobility | Self-perceived Functional Handicap (VHI) | R = -0.53 |
| Singh et al., 2010 (3)^#^ | FA | 22 | Intelligibility | FARS | R = -0.58 |
| Vogel et al. 2023 (5)^a^ | FA | 132 | Acoustic composite | Intelligibility | MAE = 0.4 (5-point scale) |
| Vogel et al. 2023 (5)^a^ | FA | 132 | Acoustic composite | Naturalness | MAE = 0.3 (5-point scale) |
| Vogel et al. 2023 (5)^a^ | FA | 132 | Acoustic composite | FARS | MAE = 15.45 (169-point scale) |
| Rosen et al. 2012 (6)^a^ | FA | 37 | Acoustic composite | FARS | R = 0.81 |
| Borel et al., 2019(4)^b^ | FA | 40 | Maximum phonation time | GAA1 | R = -0.63 |
| Borel et al., 2019 (4) ^b^ | FA | 40 | Maximum phonation time | SARA | R = -0.55 |
| Wolf et al., 2017 (7) | SCA3 | 31 | Perceived altered speech rhythm | Age at onset | R = -0.708 |
| Wolf et al., 2017 (7) | SCA3 | 31 | Perceived altered speech rhythm | CAG | R = -0.570 |
| Wolf et al., 2017 (7) | SCA3 | 31 | Perceived consonant imprecision | CAG | R = 0.593 |
| Poole et al., 2015 (8) | FA | 37 | Perceived hypernasality | GAA2 | R = 0.37 |
| Wolf et al., 2017 (7) | SCA3 | 31 | Perceived hypernasality | CAG | R = 0.417 |
| Wolf et al., 2017 (7) | SCA3 | 31 | Perceived loss of variability of pitch | Age at onset | R = -0.608 |
| Wolf et al., 2017 (7) | SCA3 | 31 | Perceived sound prolongation | CAG | R = 0.443 |
| Folker et al., 2010 (9) | FA | 7 | Perceived severity of dysarthria | FARS | R = 0.517 |
| Folker et al., 2010 (9) | FA | 7 | Perceived severity of dysarthria | Disease duration | R = 0.515 |
| Folker at al., 2010 (9) | FA | 7 | Perceived severity of dysarthria | GAA2 | R = 0.36 |
| Borel et al., 2019 (4) | FA | 40 | [badego] syllable repetition rate (DDK) | SARA | R = -0.58 |
| Singh et al., 2010 (10) | FA | 22 | [pata] / [pataka] syllable repetition rate (DDK) | FARS | R = -0.75 / -0.81 |
| Wolf et al., 2017 (7) | SCA3 | 31 | Perceived slow rate of [pa] / [ta] / [ka] / [a] / [pataka] syllables repetition (DDK) | CAG | R = 0.419 |
| Vogel et al., 2020 (11) | SCA2 | 30 | Perturbation of DDK period | CAG | R = 0.62 |
| Vogel et al., 2020 (11) | SCA2 | 30 | Perturbation of DDK period | SARA | R = 0.52 |
| Schmitz Hübsch et al 2010 (12) | SCA 1, 2, 3, 6 | 43 SCA1, 61 SCA2, 37 SCA3, 30 SCA6 | Syllable repetition frequency  Average interval length (COV) | SARA  SARA item 4  SARA  SARA item 4 | R = −0.680  R = −0.710  R = 0.444  R = 0.333 |
| Vogel et al., 2017 (13) | POLG-A | 14 | Self-evaluation of communication (subscale of SWAL-QOL) | SARA | R = -0.644 |
| Vogel et al., 2017 (14) | FA | 36 | Text reading (speech  sample duration) | FARS | R = 0.548 |
| Vogel et al., 2017 (14) | FA | 36 | Text reading (speech  sample duration) | Age at onset | R = -0.537 |
| Vogel et al., 2017 (14) | FA | 36 | Text reading (speech  sample duration) | Disease duration | R = 0.477 |
| Vogel et al., 2017 (13) | POLG-A | 14 | Variability in speech rate | Disease duration | R = 0.604 |
| Gomez-Coello et al., 2017 (15) | SCA7 | 33 | Voice perturbation | INAS | R = 0.41 (jitter) and R = 0.454 (shimmer) |
| Vogel et al., 2017 (13) | POLG-A | 14 | Vowel distortion | SARA | R = 0.616 |

^a^ Includes longitudinal data >2 years; ^b^ Includes longitudinal data =1 year; CAG= repeat expansion number cytosine-adenine-guanine; CCFS = Composite Cerebellar Functional Severity Score; DDK= diadochokinetic tasks; FARS = Friedreich Ataxia Rating Scale (16); GAA1= expansion on smaller allele of FXN; GAA2 = expansion on larger allele of FXN; INAS = Inventory of Non-Ataxia Signs; SARA = Assessment and Rating of Ataxia (17); VHI = Voice Handicap Index; FA = Friedreich ataxia; SCA = spinocerebellar ataxia; POLG-A = Mutations in the nuclear-encoded mitochondrial DNA polymerase gamma - associated ataxia # Response to study (18)

References

1. Eigentler A, Rhomberg J, Nachbauer W, Ritzer I, Poewe W, Boesch S. The scale for the assessment and rating of ataxia correlates with dysarthria assessment in Friedreich's ataxia. Journal of Neurology. 2011;259(3):420-6.

2. Vogel AP, Rommel N, Oettinger A, Stoll LH, Kraus E-M, Gagnon C, et al. Coordination and timing deficits in speech and swallowing in autosomal recessive spastic ataxia of Charlevoix–Saguenay (ARSACS). Journal of Neurology. 2018;265(9):2060-70.

3. Singh A, Epstein E, Myers LM, Farmer JM, Lynch DR. Clinical measures of dysarthria in Friedreich Ataxia. Movement Disorders. 2010.

4. Borel S, Gatignol P, Smail M, Monin ML, Ewenczyk C, Bouccara D, et al. Oral mobility reflects rate of progression in advanced Friedreich's ataxia. Ann Clin Transl Neurol. 2019;6(9):1888-92.

5. Vogel AP, Maruff P, Reece H, Carter H, Tai G, Schultz BG, et al. Clinically meaningful metrics of speech in neurodegenerative disease: Quantification of speech intelligibility and naturalness in ataxia. medRxiv. 2023:2023.03.28.23287878.

6. Rosen KM, Folker JE, Vogel AP, Corben LA, Murdoch BE, Delatycki MB. Longitudinal change in dysarthria associated with Friedreich ataxia: a potential clinical endpoint. Journal of Neurology. 2012;259(11):2471-7.

7. Wolf AE, Mourão L, Jr. França MC, Machado Júnior AJ, Crespo AN. Phonoarticulation in spinocerebellar ataxia type 3. European Archives of Oto-Rhino-Laryngology. 2017;274(2):1139-45.

8. Poole ML, Wee JS, Folker JE, Corben LA, Delatycki MB, Vogel AP. Nasality in Friedreich ataxia. Clinical Linguistics and Phonetics. 2015;29(1):46-58.

9. Folker JE, Murdoch BE, Cahill LM, Delatycki MB, Corben LA, Vogel AP. Dysarthria in Friedreich’s ataxia: a perceptual analysis. Folia Phoniatrica et Logopaedia. 2010;62(3):97-103.

10. Basu D, Jhirwal OMP, Singh J, Kumar S, Mattoo SK. Inhalant abuse by adolescents: A new challenge for Indian physicians. Indian Journal of Medical Sciences. 2004;58(6):245-49.

11. Vogel AP, Magee M, Torres-Vega R, Medrano-Montero J, Cyngler MP, Kruse M, et al. Features of speech and swallowing dysfunction in pre-ataxic spinocerebellar ataxia type 2. Neurology. 2020;95(2):e194-e205.

12. Schmitz-Hubsch T, Fimmers R, Rakowicz M, Rola R, Zdzienicka E, Fancellu R, et al. Responsiveness of different rating instruments in spinocerebellar ataxia patients. Neurology. 2010;74(8):678-84.

13. Vogel AP, Rommel N, Oettinger A, Horger M, Krumm P, Kraus E-M, et al. Speech and swallowing abnormalities in adults with POLG associated ataxia (POLG-A). Mitochondrion. 2017;37:1--7.

14. Vogel AP, Wardrop MI, Folker JE, Synofzik M, Corben LA, Delatycki MB, et al. Voice in Friedreich Ataxia. Journal of Voice. 2017;31(2):243.e9-.e19.

15. Gómez-Coello A, Valadez-Jiménez VM, Cisneros B, Carrillo-Mora P, Parra-Cárdenas M, Hernández-Hernández O, et al. Voice Alterations in Patients With Spinocerebellar Ataxia Type 7 (SCA7): Clinical-Genetic Correlations. Journal of Voice.

16. Lynch DR, Farmer JM, Tsou AY, Perlman S, Subramony SH, Gomez CM, et al. Measuring Friedreich ataxia: complementary features of examination and performance measures. Neurology. 2006;66(11):1711-6.

17. Accolla EA, Herrojo Ruiz M, Horn A, Schneider G-H, Schmitz-Hübsch T, Draganski B, et al. Brain networks modulated by subthalamic nucleus deep brain stimulation. Brain. 2016;139(9):2503-15.

18. Vogel AP, Morgan AT. Assessment of impairment or monitoring change in Friedreich ataxia. Movement Disorders. 2010;25(11):1753-4.
